# Comparing the effect of three levels of end-expiratory pressure during facemask ventilation on gastric insufflation in patients with obesity: a randomized controlled trial

**DOI:** 10.1007/s00540-025-03531-9

**Published:** 2025-06-30

**Authors:** Maha Mostafa, Ahmed Hasanin, Mohamed M. Zakaria, Hamza Kandel, Walid Hamimy, Ayman Abougabal, Mamdouh M. Elshal

**Affiliations:** 1 Department of Anesthesia and Critical Care Medicine, Cairo University, Cairo, Egypt; 2 Department of Anesthesia and Critical Care Medicine, National Cancer Institute, Cairo University, Cairo, Egypt

**Keywords:** Facemask ventilation, Gastric insufflation, PEEP, Gastric ultrasound, Obese

## Abstract

**Background:**

We compared the effect of three levels of end-expiratory pressure on the incidence of gastric insufflation during face-mask ventilation in patients with obesity.

**Methods:**

This randomized controlled trial included adult obese patients undergoing elective non-cardiac surgery under general anesthesia with neuromuscular blockade. Patients were randomized to receive either zero-end-expiratory pressure (ZEEP group), 4-cmH_2_O positive end-expiratory pressure (PEEP) (low-PEEP group), or 8-cmH_2_O PEEP (high-PEEP group) during volume-controlled mask ventilation. Gastric antral cross-sectional area (CSA) was assessed using ultrasonography before induction of anesthesia and after intubation. The percentage of change (delta) in the CSA was calculated and gastric insufflation was considered significant when the delta CSA was > 30%. The primary outcome was the incidence of gastric insufflation. Secondary outcomes were antral CSA before induction of anesthesia and after intubation in addition to ventilatory variables (end-tidal CO_2_, peak airway pressure, and tidal volume) during face-mask ventilation.

**Results:**

We analyzed data from 160 patients. The antral CSA increased after intubation in all groups. The incidence of gastric insufflation was higher in the high-PEEP group (32/54[59%]) than that in the ZEEP group (6/52[12%]) and low-PEEP group (15/54[28%]). Delta CSA, antral CSA after intubation, and incidence of gastric insufflation were not significantly different between the ZEEP and low-PEEP groups. Ventilatory variables were comparable between the groups.

**Conclusion:**

In obese paralyzed patients, gastric insufflation can occur during face-mask ventilation whatever the level of end-expiratory pressure; however, the use of ZEEP or 4-cmH_2_O PEEP was associated with lower incidence of gastric insufflation compared to 8-cmH_2_O PEEP.

**Clinical trial registration:**

Clinical trial registration at clinicaltrials.gov NCT05979129. https://classic.clinicaltrials.gov/ct2/show/NCT05979129

## Introduction

Pulmonary aspiration of gastric contents is a severe and potentially life-threatening complication of general anesthesia [1]. Key predisposing factors for gastric regurgitation and pulmonary aspiration include increased gastric content and reduced lower esophageal sphincter pressure [2]. Gastric insufflation during facemask ventilation can distend the stomach, increasing the risk of regurgitation. This insufflation occurs when airway pressure exceeds upper esophageal sphincter pressure and regurgitation occurs when gastric pressure exceeds the lower esophageal sphincter pressure [3].

Evidence from non-obese populations suggests that maintaining a peak airway pressure below 15 cmH_2_O during facemask ventilation achieves a balance between attaining effective ventilation and minimizing the risk of gastric insufflation [4–6]. However, patients with obesity present a unique challenge for their compromised respiratory mechanics which further deteriorate following anesthesia induction and neuromuscular blockade [7]. As a result, achieving adequate ventilation in obese patients often necessitates higher inspiratory pressures.

The use of positive end-expiratory pressure (PEEP) during facemask ventilation is recommended for obese patients to prevent anesthesia-induced atelectasis and hypoxia [8, 9] however, a study in non-obese patients showed that the application of PEEP of 10 cmH_2_O PEEP during facemask ventilation increased the risk of gastric insufflation [10]. Currently, there is a lack of data about the impact of lower levels of PEEP on the risk of gastric insufflation, particularly in obese patients.

This study aims to evaluate the effect of three levels of end-expiratory pressure during facemask ventilation on the risk of gastric insufflation in obese patients. We hypothesized that the application of high PEEP (8 cmH_2_O) would result in a greater risk of gastric insufflation compared to low PEEP (4 cmH_2_O) or zero end-expiratory pressure (ZEEP).

## Patients and methods

This randomized controlled trial was conducted at Cairo University Hospital between August 2023 and April 2024, after institutional research ethics committee approval (MD-65–2022). Patients’ recruitment started after registration at clinicaltrials.gov (NCT05979129, date: July 30, 2023). Written informed consent was obtained from the patient before enrollment.

Participants were adult patients (18–60 years), American society of anesthesiologists physical status II–III, with body mass index > 35 kg/m^2^, scheduled for elective non-cardiac surgery under general anesthesia with neuromuscular blockade. Exclusion criteria were suspected of having difficult mask ventilation [11] or intubation, increased risk of aspiration, history of esophageal reflux, and pregnancy.

Online random sequence was generated to allocate the patients to the study group in a 1:1:1 ratio (https://www.graphpad.com/quickcalcs/randomize1/). The group assignment according to the randomization number was concealed inside sequentially numbered opaque envelopes. A research assistant was responsible for opening the envelopes and informing the attending anesthetist about the level of PEEP to be used during the facemask ventilation. The research assistant had no further involvement in the study.

Patients were instructed to fast for 6 h for solids and 2 h for clear fluid. Before induction of anesthesia, routine monitors (electrocardiogram, pulse oximetry, and non-invasive blood pressure monitor) were applied and an intravenous line was secured. End-tidal CO_2_ monitoring started after the induction of general anesthesia and starting facemask ventilation. All patients were positioned in the ramped position (achieved by elevation of the head and shoulders until achieving alignment of sternal notch with external auditory meatus). Preoxygenation was achieved by asking the patient to breathe through a facemask with a fraction of inspired oxygen (FiO_2)_ of 0.8 and without PEEP for three minutes. Induction of anesthesia was achieved using 2 mcg/kg fentanyl (lean body weight) and 2 mg/kg propofol (lean body weight). Boluses of 0.25 mg/kg of propofol were given if loss of consciousness was not achieved by the initial bolus. After loss of consciousness, 0.5 mg/kg atracurium (ideal body weight) was administered. Facemask ventilation was achieved by double hand jaw thrust head tilt maneuver using appropriately sized facemask (size: 4, 5) and with the aid of oropharyngeal airway (Guedel airway size: 3, 4). The participant received volume-controlled ventilation adjusted to deliver a tidal volume of 8–10 mL/kg (ideal body weight) [4, 12], at an I:E ratio of 1:2, inspiratory pause of 0.5 s, respiratory rate of 12 breath per minute, and FiO_2_ of 0.8. Anesthesia was maintained using Maquet Flow-I anesthesia machine (Getinge AB, Gothenburg, Sweden). The patients received end-expiratory pressure during facemask ventilation according to the randomization as follows: either zero end-expiratory pressure (ZEEP group), 4-cmH_2_O PEEP (low-PEEP group), or 8-cmH_2_O PEEP (high-PEEP group). The three study groups received the planned ventilatory strategy for 120 s. Neuromuscular monitoring was not used to confirm the level of blockade before tracheal intubation. Tracheal intubation was performed 2 min after the administration of the neuromuscular blocker [13]. The tidal volume in this study was 8–10 mL/kg to compensate for the anatomical dead space (2.2 ml/kg). Furthermore, previous studies reported that a tidal volume of 6–10 mL/kg was adequate during mask ventilation. [4, 12] The assigned tidal volume for each patient was constant during the study period.

Gastric insufflation was assessed using ultrasonography by an expert operator (who was blinded to the PEEP level). The examination was performed using a 2–5 MHz transducer connected to Siemens (Acuson X300, Korea). The gastric antrum was assessed at the sagittal plane between the left lobe of the Liver and Pancreas at level of the Aorta. Gastric antral cross-sectional area (CSA) was calculated as: (longitudinal diameter) × (anteroposterior diameter) × π/4. CSA was assessed in between contractions before induction of anesthesia and after insertion of the tracheal tube [14]. The percentage of change in the CSA was calculated as follows: delta CSA = [CSA after intubation—baseline CSA]/baseline CSA X 100. Gastric insufflation was considered significant when delta CSA was > 30% [5].

A stethoscope, applied over the epigastrium, was used for intermittent gastric auscultation during facemask ventilation. Auscultation was performed by a blinded investigator at 30, 60, 90, 120 s. The presence of gastric insufflation was identified by hearing a gurgling sound.

Ventilatory variables (peripheral O_2_ saturation, tidal volume, peak airway pressure, and end-tidal CO2) and hemodynamic variables (heart rate and mean arterial pressure) were recorded at 30, 60, 90, 120 s after induction of anesthesia and after confirming tracheal intubation.

The primary outcome was the incidence of gastric insufflation by ultrasonography. Secondary outcomes were incidence of gastric insufflation, detected by auscultation; antral CSA before induction of anesthesia and after intubation; delta CSA in percentage; end-tidal CO_2_; peak airway pressure; and tidal volume (mL/kg ideal body weight). Data were obtained during facemask ventilation at 30, 60, 90, and 120 s and immediately after tracheal intubation. The incidence of bradycardia (heart rate < 60 beat/min) and hypotension (mean arterial pressure < 65 mmHg) was also reported.

### Sample size calculation

Sample size was calculated using MedCalc Statistical Software version 14.10.2 (MedCalc Software bvba, Ostend, Belgium). In a pilot study on 12 patients, the incidence of gastric insufflation after facemask ventilation with PEEP 8 cmH2O was 75%. At alpha error of 0.025 (Bonferroni correction), we calculated that 156 patients would give 80% power to detect 25% reduction in the frequency of gastric insufflation in the treatment groups. The number of envelopes was increased to 162 (54 per group) to compensate for possible dropouts.

### Statistical analysis

Statistical package for social science (SPSS) software, version 26 for Microsoft Windows (IBM. Corp., NY, USA) was used for data analysis. Categorical data are expressed as frequency (percentage) and analyzed using the chi-squared test. The relative risk for the incidence of delta CSA > 30% in relation to the high-PEEP group was calculated. Continuous data were tested for normality using the Shapiro–Wilk test and skewed data are presented as median (quartiles) while normally distributed data are presented as mean (standard deviation). Continuous un-paired data were analyzed using the one-way analysis of variance with post hoc Tukey modification (for normally distributed data) or the Kruskal–Wallis test (for skewed data). The median differences between groups were estimated using the Hodges–Lehmann estimator. All confidence levels were adjusted to 98.33% (Bonferroni-corrected) for each pairwise comparison to maintain an overall confidence level of 95%. Repeated measures (measured tidal volume, peak airway pressure, and end-tidal CO_2_) were analyzed using analysis of variance for repeated measures. Paired skewed data (antral CSA) were analyzed using the Wilcoxon signed-rank test. The effect of tidal volume, peak airway pressure, time, and level of PEEP on the primary outcome was assessed using the generalized estimating equation. The Bonferroni method was used to adjust the *p* value for multiple comparisons. A *p* value of < 0.05 was considered statistically significant.

## Results

**Fig. 1:**
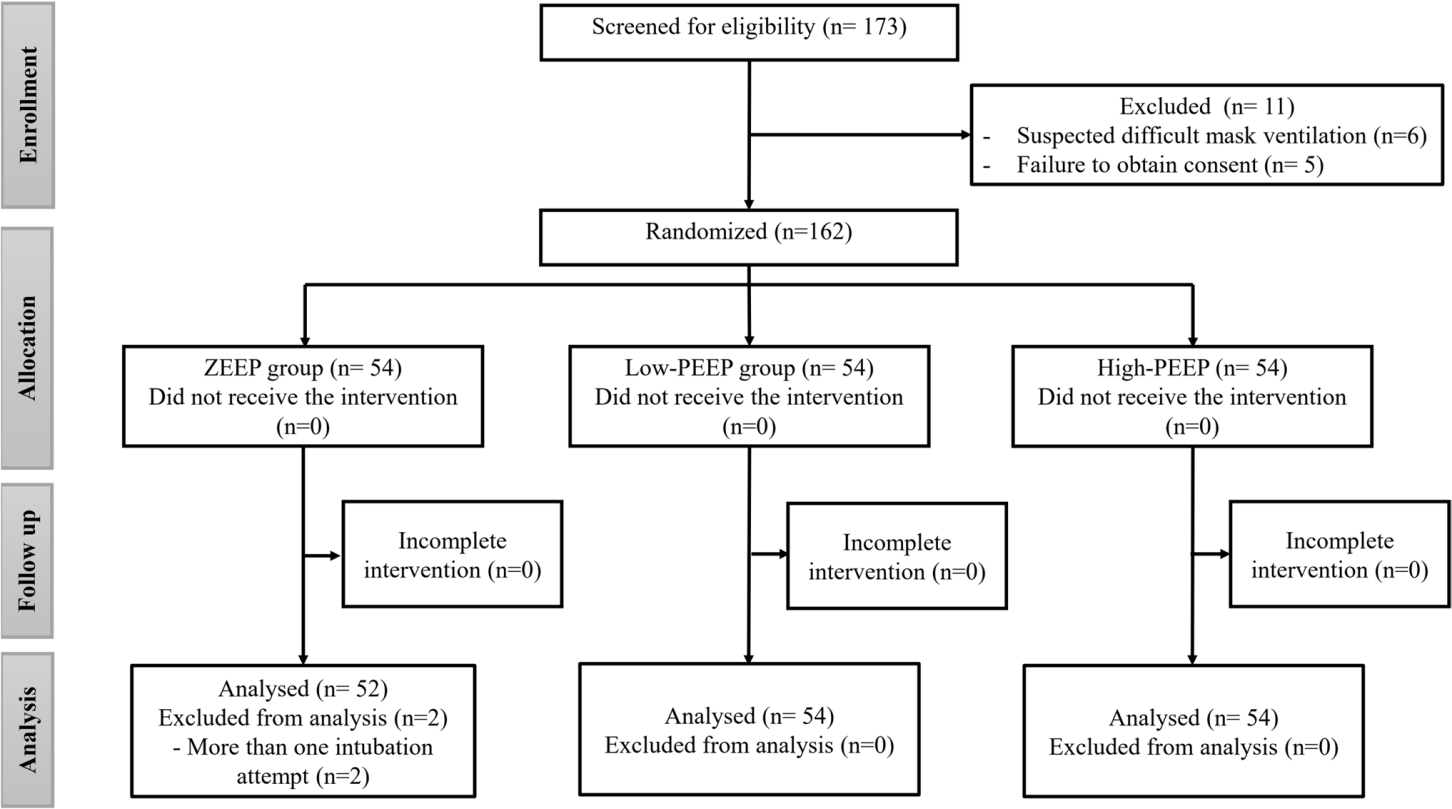
CONSORT’s flowchart. ZEEP: zero end-expiratory pressure, PEEP: positive end-expiratory pressure

One hundred and seventy-three patients were screened for eligibility. Eleven patients were excluded for not meeting the inclusion criteria, and 162 patients were randomized into one of the study’s three groups (54 patients in each group). Two patients in the ZEEP group were excluded due to repeated intubation attempts and reapplication of facemask ventilation. (Fig. 1) Demographic data and baseline characteristics were not significantly different between the study’s groups (Table 1).

**Table 1:** Demographic and baseline characteristics. Data are presented as median (quartiles) and frequency (%)

	ZEEP group (*n* = 52)	Low-PEEP group (*n* = 54)	High-PEEP group (*n* = 54)
Age (years)	37 (28, 44)	38 (31, 43)	32 (28, 44)
Male sex	9 (17%)	9 (17%)	11 (20%)
Weight (kg)	106 (98, 120)	106 (95, 115)	110 (95, 125)
Body mass index (kg.m^−2^)	39 (37, 42)	39 (37, 42)	41 (37, 44)
Comorbidity			
Smoking	7 (14%)	9 (17%)	6 (11%)
Diabetes mellitus	14 (27%)	15 (28%)	15 (28%)
Hypertension	6 (12%)	11 (20%)	11 (20%)

*ZEEP* zero end-expiratory pressure, *PEEP* positive end-expiratory pressure

The antral CSA increased after intubation in comparison to the baseline measurement in all the study groups. The delta CSA was higher in the high-PEEP group than in both the ZEEP (*p* value < 0.001) and the low-PEEP (*p* value: 0.002) groups. However, a significant difference in the antral CSA after intubation was only observed between the high-PEEP and the ZEEP groups, *p* values: 0.005 (Table 2). More patients in the high-PEEP group (32/54[59%]) had delta CSA > 30% than in the ZEEP (6/52[12%]) and the low-PEEP (15/54[28%]) groups, *p* value: < 0.001 and 0.001, respectively. The relative risk (98.3% CI) of the incidence of delta CSA > 30% in ZEEP and low-PEEP in relation to the high-PEEP group was 5.14 (2.03–13.02) and 2.13 (1.20–3.79), respectively. Also, the incidence of gastric insufflation by auscultation was higher in the high-PEEP group than the other two groups (*p* value < 0.001 for both comparisons) (Table 2).

**Table 2:** Gastric ultrasound examination and auscultation. Data are presented as median (quartiles) and frequency (%)

	ZEEP group (*n* = 52)	Low-PEEP group (*n* = 54)	High-PEEP group (*n* = 54)	*p* value
Baseline CSA (mm^2^)	483 (429, 566)	504 (439, 593)	528 (412, 659)	0.427
Range	332–722	228–1033	192–845	
Difference (98.3% CI) vs. ZEEP group		20 (−36 to 76)	34 (−32 to 100)	
Low- vs. high-PEEP group			10 (−62 to 82)	
After intubation CSA (mm^2^)	586 (503, 651)*	608 (520, 665)*	697 (537, 817)*†	0.006
Range	357–891	345–984	241–1458	
Difference (98.3% CI) vs. ZEEP group		24 (−27 to 75)	106 (23–189)	
Low- vs. high-PEEP group			80 (−3 to 163)	
Delta CSA (%)	16 (7, 24)	19 (9, 32)	32 (18, 39) †‡	< 0.001
Range	−10 to 111	−39 to 52	−26 to 84	
Difference (98.3% CI) vs. ZEEP group		3 (−4 to 10)	15 (9–21)	
Low- vs. high-PEEP group			12 (5–19)	
Incidence of delta CSA > 30%	6 (12%)	15 (28%)	32 (59%) †‡	< 0.001
Difference (98.3% CI) vs. ZEEP group		16 (−2 to 34) %	48 (29–67) %	
Low- vs. high-PEEP group			32 (10–53) %	
Positive auscultation	9 (17%)	5 (9%)	27 (50%) †‡	< 0.001
Difference (98.3% CI) vs. ZEEP group		8 (−8 to 24) %	33 (12–53) %	
Low- vs. high-PEEP group			41 (22–60) %	

*Denotes significance in relation to baseline in each group. † Denotes significance in relation to ZEEP group, ‡ Denotes significance in relation to low-PEEP group

*CI* confidence interval, *CSA* cross-sectional area. *ZEEP* zero end-expiratory pressure, *PEEP* positive end-expiratory pressure

Delta CSA, antral CSA after intubation, and number of patients with delta CSA > 30% were not significantly different between the ZEEP and the low-PEEP group (Table 2).

The generalized estimating equation model, which included tidal volume, peak airway pressure, time, and group, showed that the level of PEEP was the only variable influencing the risk of gastric insufflation.

**Fig. 2:**
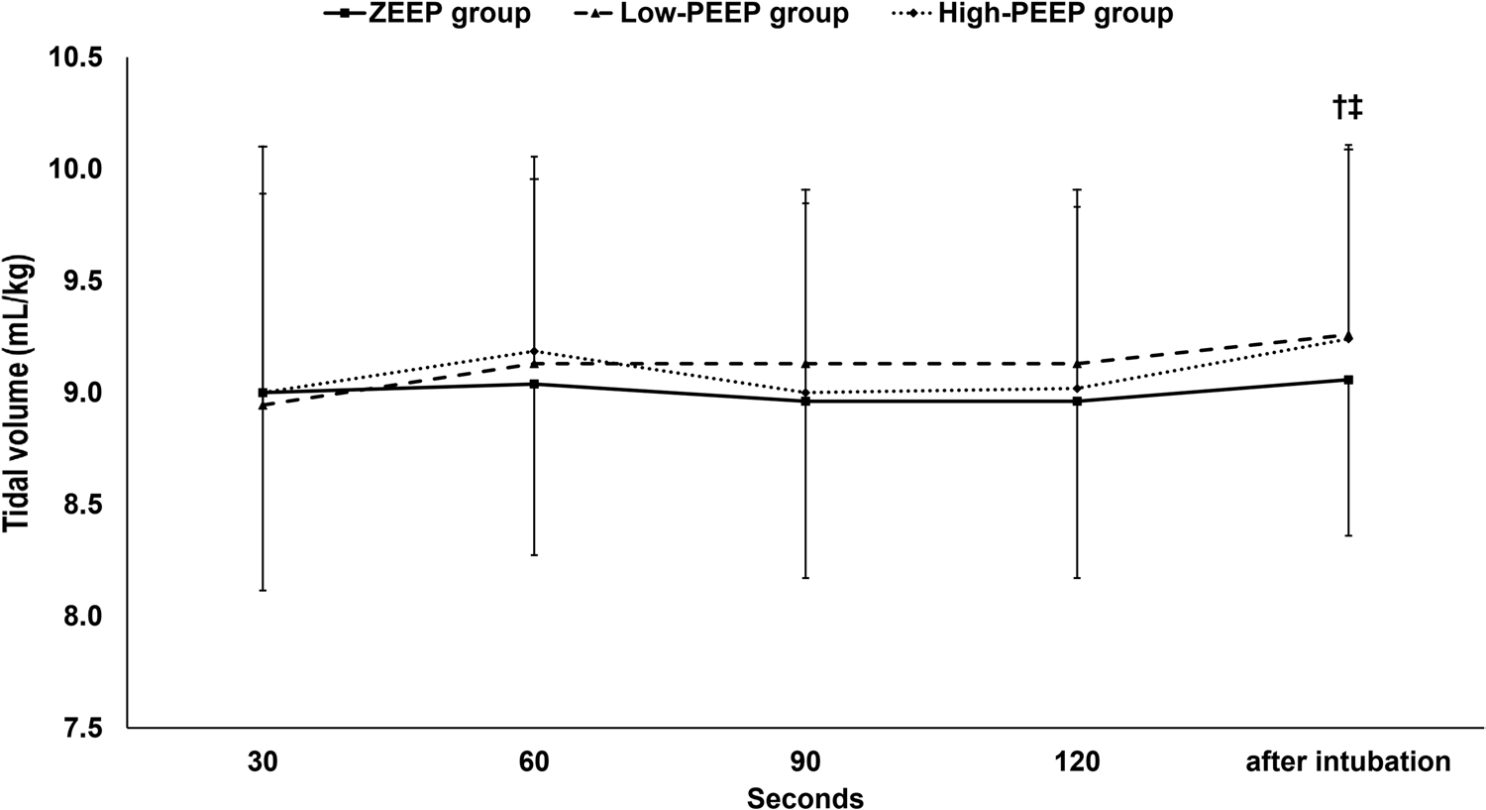
Tidal volume. Marker are means and error bars are standard deviation. † denotes significance in relation to the first reading within the low-PEEP group. *ZEEP* zero end-expiratory pressure, *PEEP* positive end-expiratory pressure

**Fig. 3:**
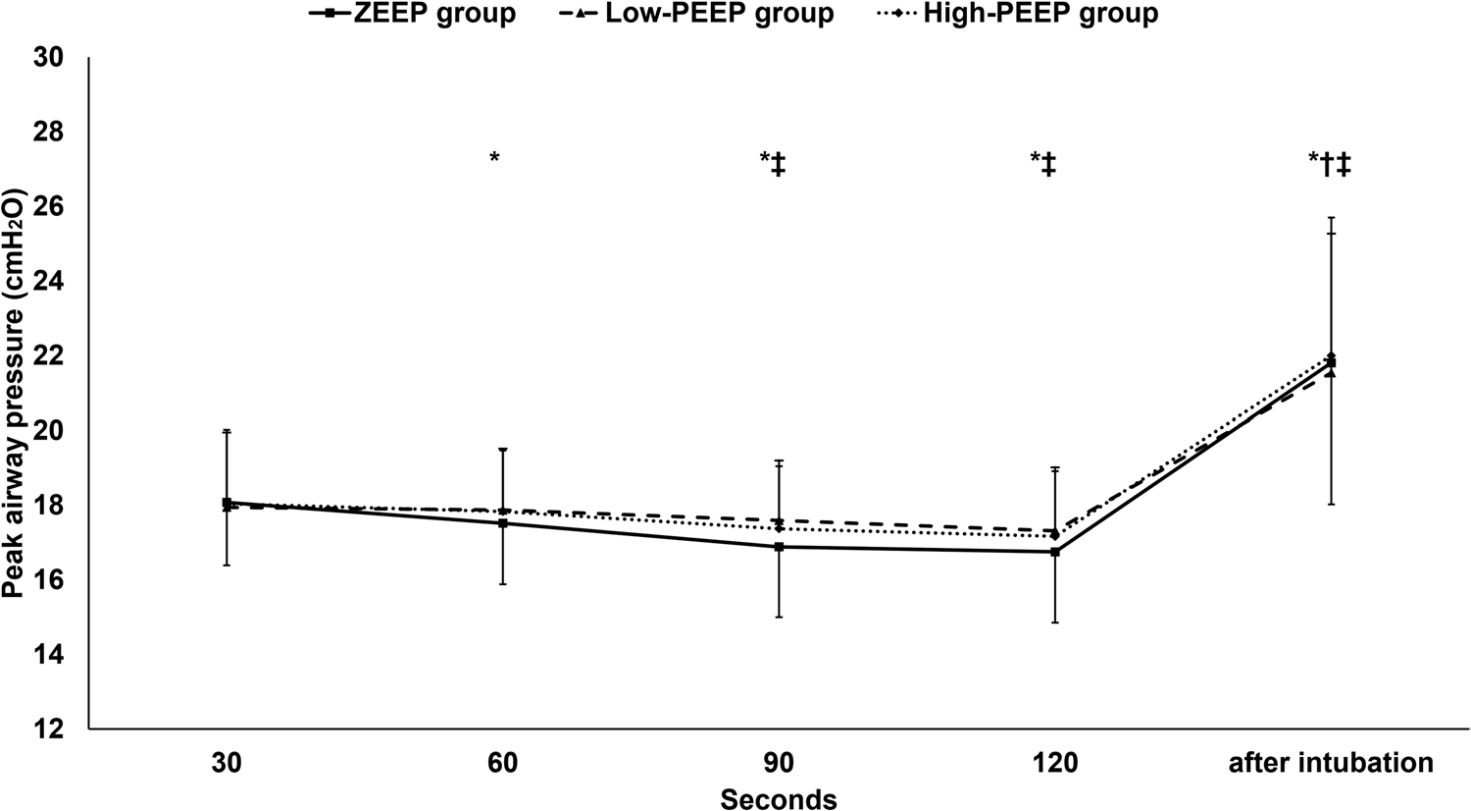
Peak airway pressure. Markers are means and error bars are standard deviations. * denotes significance in relation to the first reading within the ZEEP group, † denotes significance in relation to the first reading within the low-PEEP group, and ‡ denotes significance in relation to the first reading within the high-PEEP group. *ZEEP* zero end-expiratory pressure, *PEEP* positive end-expiratory pressure

**Fig. 4:**
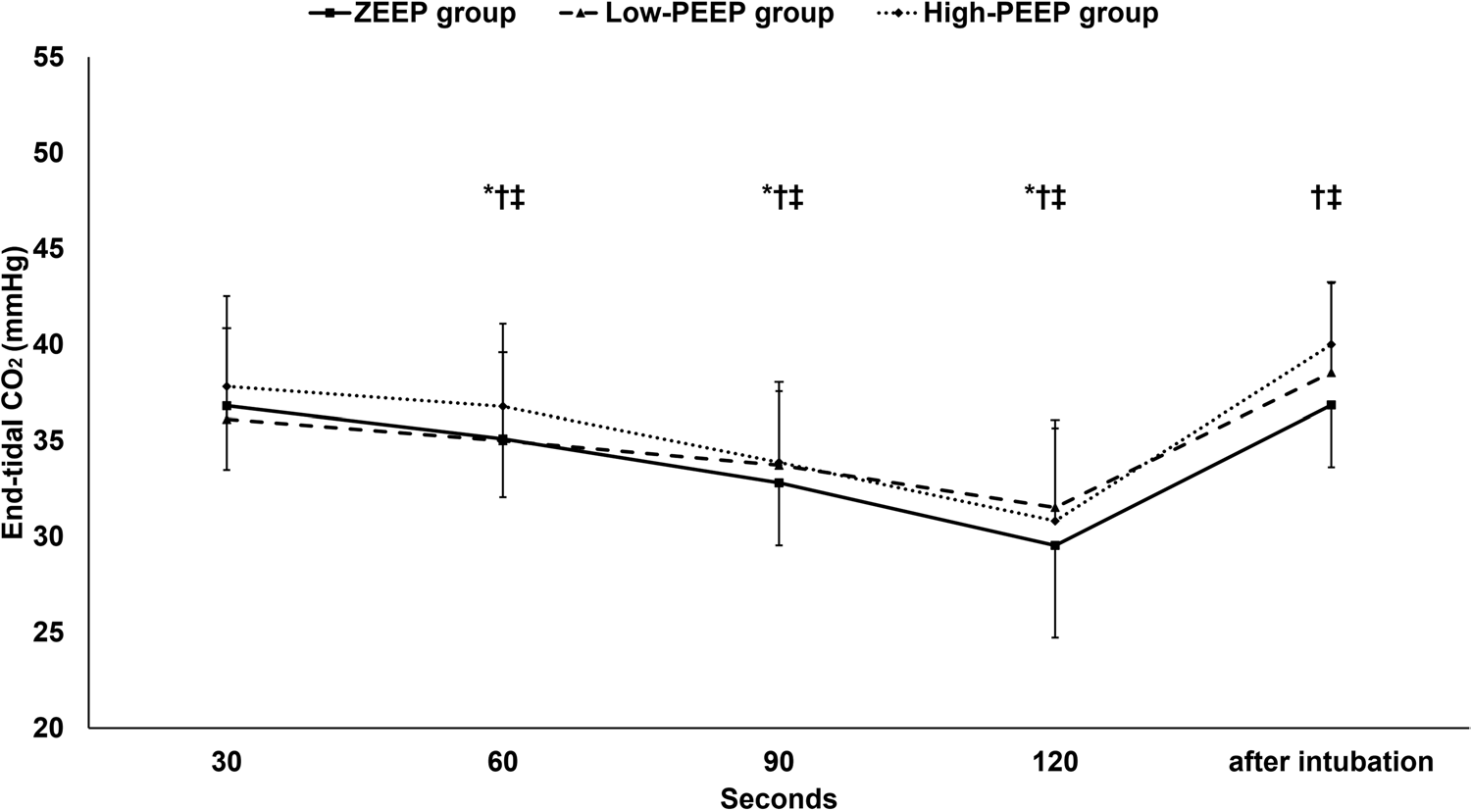
End-tidal CO2. Markers are means and error bars are standard deviations. * denotes significance in relation to the first reading within the ZEEP group, † denotes significance in relation to the first reading within the low-PEEP group, and ‡ denotes significance in relation to the first reading within the high-PEEP group. *ZEEP* zero end-expiratory pressure, *PEEP* positive end-expiratory pressure

During facemask ventilation, measured tidal volume, peak airway pressure, and end-tidal CO_2_ were not statistically different between the three groups. (Figs. 2, 3, 4) Tidal volume was maintained during facemask ventilation in the three groups. (Fig. 2) Peak airway pressure decreased with time during facemask ventilation in the ZEEP and the high-PEEP groups only. (Fig. 3) End-tidal CO_2_ decreased with time in all groups (Fig. 4).

After intubation, both measured tidal volume and end-tidal CO_2_ were higher than the initial readings in the low- and the high-PEEP groups. (Figs. 2 and 4) Peak airway pressure and measured tidal volume did not significantly differ between the groups, but the end-tidal CO_2_ was significantly higher in the high-PEEP group compared to the ZEEP group.

The average driving pressure during mask ventilation was the lowest in the high-PEEP group followed by low-PEEP group, then ZEEP group (mean [standard deviation]: 9.7 [1.3], 13.8 [1.2], and 17.4 [1.3] cmH_2_O, respectively; *p* value < 0.001). None of the included patients had SpO_2_ < 97%.

The incidence of bradycardia (1 [2%] in ZEEP, 4 [7%] in low-PEEP and 1 [2%] in high-PEEP) and hypotension (3 [6%] in ZEEP, 2 [4%] in low-PEEP, and 4 [7%] in high-PEEP) was not significantly different between the groups, *p* value: 0.221, 0.704.

## Discussion

In this study, we evaluated the effect of different levels of PEEP during facemask ventilation on gastric insufflation using ultrasonography in paralyzed obese patients. We report that the three levels of end-expiratory pressure produced adequate minute ventilation, and the gastric antral CSA increased after facemask ventilation with or without PEEP. The incidence of significant gastric insufflation was the highest after application of 8-cmH_2_O PEEP compared to either ZEEP or 4 cmH_2_O PEEP without significant difference between the two later pressures.

Gastric insufflation during facemask ventilation is attributed to the reduction of upper and lower esophageal sphincters’ tone after induction of anesthesia which allows gas passage during positive pressure ventilation. This could explain the occurrence of gastric insufflation in all groups. However, the mechanism by which the PEEP increased the risk of gastric insufflation at the same peak airway pressure remains unclear. Previous manometric and impedance study of the esophagus during facemask ventilation showed that the pressures of the upper and lower esophageal sphincters did not significantly differ with or without the application of PEEP, and that gastric insufflation occurred exclusively during the inspiratory phase [10]. This suggests that the phenomenon cannot be fully explained by the effect of PEEP on the lower esophageal sphincter. However, only a PEEP of 10 cmH_2_O had been previously studied, and it was associated with an increased risk of gastric insufflation at lower inspiratory pressures compared to ventilation without PEEP [10]; therefore, we designed this study to assess the effect of lower PEEP levels on the risk of gastric insufflation. In this study, we observed gastric insufflation even with lower PEEP levels which may also exhibit a dose-dependent relationship. This finding highlights the need for additional research to better understand this interaction.

The international consensus for protective lung strategy during surgery recommended the use of PEEP instantly after induction of anesthesia and during facemask ventilation [8]. However, it is unclear whether this recommendation applies to obese patients or not. Furthermore, data regarding the optimum PEEP during mask ventilation in patients with obesity are lacking and no definitive PEEP value is recommended. The current study is the first, to the best of our knowledge, to assess the effect of different end-expiratory pressure levels on ventilation and gastric insufflation during mask ventilation in patients with obesity. A previous study was conducted in non-paralyzed non-obese population [10]; Cajander et al. [10] had reported that application of 10 cmH_2_O PEEP during facemask ventilation increased the risk of gastric insufflation in comparison to no-PEEP and this is in line with our findings. However, Cajander et al. reported that gastric insufflation started at peak inspiratory pressure of 25 cmH_2_O without PEEP; and at 20 cmH_2_O with 10 cmH_2_O PEEP. While in the current study, the peak inspiratory pressure was ≤ 20 cmH_2_O at all time points; nevertheless, gastric insufflation occurred regardless of the level of PEEP. This discrepancy between our findings and Cajander et al.’s study could be due to several differences between the two studies. First, we included obese paralyzed patients who have lesser lower esophageal sphincter tone than non-obese patients [15] which would be further reduced by neuromuscular blockers [16], facilitating more gas insufflation into the stomach. Second, the method of gastric evaluation (gastric ultrasound in our study vs high-resolution manometry and impedance catheter in Cajandar et al. study). Third, the use of different ventilation modes, Cajander et al. used pressure-controlled ventilation while in this study we used volume-controlled ventilation. Volume-controlled ventilation could result in a higher peak airway pressure than a pressure-controlled ventilation for the same tidal volume. However, we believe that the use of volume-controlled ventilation is more appropriate in this study since applying fixed inspiratory pressure exposes the patients to either over- or under-ventilation [4, 5, 10, 17]. Furthermore, patients with obesity are characterized by a reduced lung compliance and increased airway resistance [7]. Thus, the optimum ventilating pressure could be highly variable. Future studies are needed to evaluate the adequacy of ventilation and risk of gastric insufflation using different modes of ventilation in obese patients.

We observed a progressive decrease in end-tidal CO_2_ during mask ventilation at all levels of end-expiratory pressure. After intubation, end-tidal CO_2_ increased in the low- and the high-PEEP groups but remained unchanged in the ZEEP group despite maintaining the same minute ventilation. Notably, during mask ventilation, end-tidal CO_2_ might not reliably reflect PaCO_2_ and this might suggest that the changes in the end-tidal CO_2_ values do not reflect the actual ventilation status [18]. After intubation, improved airway control likely led to more stable ventilation, better alveolar recruitment, and enhanced CO_2_ clearance; and this might explain the increased end-tidal CO_2_ at this measurement point in the low- and the high-PEEP groups.

In the current study, we used two methods to assess gastric insufflation. First, epigastric auscultation during facemask ventilation. This method is simple with high sensitivity and specificity for small amount of gas insufflation, as small as 4 mL [19]; however, its use in obese patients might be limited due to the thick abdominal wall. The second method was measurement of the gastric antral CSA using ultrasonography. This method offers simple and objective assessment of the extent of gastric insufflation. We used a 30% increase in the CSA to define gastric insufflation; this value was previously used in non-obese population [5] and was originally extracted from a study by Bouvet et al. [4]. Bouvet et al. used the comet-tail sign in ultrasonography to identify gastric insufflation during facemask ventilation in real time. In Bouvet et al. study, the average change in CSA was < 30% in patients with negative comet-tail sign and > 30% in patients with positive comet-tail sign [4]. Therefore, this cut-off value was used as a surrogate of real-time assessment since it is not feasible to apply auscultation and ultrasonography simultaneously during facemask ventilation. We found that more patients were classified positive for gastric insufflation by ultrasonography than auscultation. This is probably due to the subjective nature of auscultation method which reduces its accuracy especially in obese patients with thick abdominal wall.

The recommended mode for mechanical ventilation in obese patients is volume-controlled mode with a tidal volume ≈ 8 mL/kg plus PEEP, without a definitive value of the end-expiratory pressure [7, 20]. It is also recommended to deliver oxygen during elective intubation by providing pressure support and PEEP to recruit the alveoli and minimize atelectasis, which is common after induction of anesthesia in this population and consequently prolongs the non-hypoxic apnea time [7]. However, there is no clear recommendation for the best way to avoid gastric insufflation in the absence of tracheal tube. Our study showed that the use of higher PEEP produced lesser driving pressure compared to lower PEEP, and this is explained by the improved lung compliance and the positive transpulmonary pressure [7]. The use of PEEP in patients with obesity antagonizes the impact of the abdominal as well as chest wall load [7]. The benefit of improved lung compliance with the use of higher PEEP during facemask ventilation was unfortunately associated with increased gastric insufflation even with similar peak airway pressure.

There is an increasing number of patients with obesity who are presenting for surgery (bariatric or non-bariatric) [21]. Patients with obesity are high-risk patients that represent a challenge for the anesthetist due to associated comorbidities, increased risk of difficult airway and impaired respiratory mechanics [21]. Patients with obesity are at increased risk of rapid oxygen desaturation after induction of anesthesia in comparison to non-obese patients due to reduced functional residual capacity and oxygen reserve in addition to the higher metabolic rate and oxygen consumption [21]. Therefore, the use of PEEP during facemask ventilation had been suggested in patients with obesity to provide the safest possible apnea time [22]. On the other hand, gastric insufflation during facemask ventilation could increase the gastric pressure which in turn could increase the risk of regurgitation and aspiration of gastric content.

Our findings indicates that gastric insufflation could occur during facemask ventilation in obese patients whatever the level of PEEP with the highest risk at PEEP 8 cmH_2_O. Therefore, we suggest the use of other alternatives for improving oxygenation in these patients such as the use of continuous positive airway pressure before induction of anesthesia, where the tone of the esophageal sphincter is still preserved. After induction of anesthesia, we assume that the decision should be individualized according to the risk of hypoxia and aspiration. Application of PEEP should be cautious and should not exceed 4 cmH_2_O and better avoided in  patients with high-risk of aspiration.

This study has some limitations. It was conducted in a single center. We excluded patients with suspected difficult airway or at risk of aspiration as these patients would have different techniques of airway management. Oral airway was used in all patients which might negate the potential advantage of PEEP in maintaining a patent airway during obstruction and subsequently ventilating patients at lower airway pressures compared to ZEEP. In clinical practice, anesthetists would address airway obstructions with measures such as application of continuous positive airway pressure (CPAP) or insertion of an oral airway [8]. Therefore, we used an oral airway to standardize mask ventilation. Additionally, the primary outcome is a surrogate measure of the true outcome, which is the pulmonary aspiration of gastric contents.

## Conclusion

In obese paralyzed patients, gastric insufflation can occur during facemask ventilation regardless of the presence or absence of PEEP. However, the use of ZEEP or 4-cmH_2_O PEEP was associated with lower incidence of gastric insufflation compared to 8-cmH_2_O PEEP without significant difference between the two former pressure levels. All levels of end-expiratory pressure produced adequate minute ventilation and similar peak airway pressure.

## Data Availability

The datasets used and/or analyzed during the current study are available from the corresponding author upon reasonable request.
